# Agent-based null models for examining experimental social interaction networks

**DOI:** 10.1038/s41598-023-32295-z

**Published:** 2023-03-31

**Authors:** Susan C. Fennell, James P. Gleeson, Michael Quayle, Kevin Durrheim, Kevin Burke

**Affiliations:** 1 MACSI, Department of Mathematics and Statistics, University of Limerick, Limerick, Ireland; 2 Department of Psychology, University of Limerick, Limerick, Ireland; 3 Department of Psychology, University of KwaZulu-Natal, Pietermaritzburg, KwaZulu-Natal South Africa; 4 Department of Psychology, University of Johannesburg, Johannesburg, South Africa

**Keywords:** Psychology, Mathematics and computing

## Abstract

We consider the analysis of temporal data arising from online interactive social experiments, which is complicated by the fact that classical independence assumptions about the observations are not satisfied. Therefore, we propose an approach that compares the output of a fitted (linear) model from the observed interaction data to that generated by an assumed agent-based null model. This allows us to discover, for example, the extent to which the structure of social interactions differs from that of random interactions. Moreover, we provide network visualisations that identify the extent of ingroup favouritism and reciprocity as well as particular individuals whose behaviour differs markedly from the norm. We specifically consider experimental data collected via the novel Virtual Interaction APPLication (VIAPPL). We find that ingroup favouritism and reciprocity are present in social interactions observed on this platform, and that these behaviours strengthen over time. Note that, while our proposed methodology was developed with VIAPPL in mind, its potential usage extends to any type of social interaction data.

## Introduction

Social interaction is a driving force in shaping opinions, beliefs, and behaviours. Understanding how the emergence and evolution of social structures, such as norms and identities, depends on social interaction is an important avenue of research in social psychology, sociology, economics and related disciplines^1–6^. While there has been great interest in understanding the emergence through social interaction of a variety of attitudes and behaviors, such as norms, social influence, reciprocity, group cohesion and so on^7–9^, interaction has been difficult to observe in experimentally controlled environments. This obstacle is being addressed by several groups developing software platforms for running experiments in networks with interaction, including VIAPPL (which allows for the study of emergent behaviour^10–12^; http://www.viappl.org), Breadboard (which focuses more explicitly on experimental manipulation of network wiring^13,14^; http://breadboard.yale.edu) and HuGoS (which explores human swarming behaviour^15^) amongst others. Of particular interest is the nature of participant-to-participant and group-level interactions over time. Such interactions generate temporal network data, i.e., networks where individuals are linked at a given time point if they have interacted, and these networks may be weighted by the frequency of the interactions. While the key reason to study these social systems is the expectation that participants’ behaviours are both socially and temporally dependent, the presence of such dependence is in clear violation of the independence assumptions of many classical statistical procedures such as linear regression, i.e., estimated standard errors (and, hence, p values) will typically be invalid. (Notwithstanding this fact, it has been found that linear regression may still be appropriate for some forms of network data^16^.)

As a motivating example of temporal social interaction data used throughout this article, we consider experimental data collected using VIAPPL, the Virtual Interaction APPLication (see http://www.viappl.org). VIAPPL is a novel software environment that enables controlled social interaction experiments, allowing for the study of how social structures emerge over time though social interaction. Participants, or players, in these experiments appear as avatars in a virtual “game”, wherein they are allocated to a group, and they interact with each other by exchanging “tokens” over a number of rounds; these tokens can be described to participants as points or may have real monetary value, depending on the aims of the experiment, e.g., to invoke group-level competitiveness or individual-level self-interest. However, the game is not a competition per se (i.e., there is no “winner”), and players are not given any expectations about the game beyond being informed that they will be placed in groups and exchange tokens with each other. Games can be initialised with various experimental manipulations, such as limiting token exchange to one per player per round, or making one group “rich” and another “poor”, and these manipulations impose specific constraints on the sample space of networks that can be realised.

In this paper, we consider games analogous to Tajfel and colleagues’ minimal group studies, which showed that allocating a participant to an arbitrary group is sufficient to induce ingroup bias^17^. The VIAPPL games maintain the simplicity of these classical minimal group studies, but add social interaction over a series of rounds. In these designs, players are randomly assigned to one of two groups at the beginning of the experiment and only interact/communicate through token exchange (not, for example, face-to-face or through a chat function). Specifically, each round, players select the avatar of a player to whom they want to allocate one of their tokens; they may also select themselves, which may be an attempt at preserving their token balance or simply a decision not to engage with others. The round concludes when all players have made a selection, at which point, they are presented with a network diagram of all individual interactions from the previous round, where group membership is indicated. Ultimately, players use this information to inform their decisions for the next round, and, in this way, their interactions influence behaviour and shape norms in a given game. The output of this experiment is a time-dependent (directed) weighted network whose links describe the number of times each individual has interacted with each other up to each round. While we have described a type of VIAPPL game here, of course this general setup of individuals from different groups interacting over time is observable throughout society (albeit without experimental control).

As previously mentioned, the use of classical statistical techniques (e.g., t-test or linear regression^18,19^) for temporal social network data is typically not appropriate. A key challenge is that observations (links in the network) are not likely to be independent. This is not only due to the fact that a given individual is measured at multiple time points, but also due to the (potentially complex) dependence structures that can exist in network data^16^, i.e., when deciding on their next action, an individual may be influenced by the actions of their neighbours, their neighbours’ neighbours, and so on. More specific to the VIAPPL games we consider here is that each player carries out one interaction per round, which leads to a constraint on the total number of interactions, and, hence, the possible network structures that can be observed. (However, similar constraints may exist in other settings due to limitations on time, resources, or attention span for example.) Existing network modelling approaches such as the stochastic actor-oriented model (SAOM)^20^ and the Temporal Exponential Random Graph Model (TERGM)^21^ are not well-suited to our setting since they are not designed to capture the aforementioned experimental constraints. Indeed, SAOM has been found to be sensitive to the assumptions of its underlying generative model^22^ and, more generally, there is ongoing debate on the merits of these approaches (SAOM and TERGM) in different circumstances^23^.

We introduce a general approach for analyzing network data in applications where the rules for the generation process are well defined, which involves the use of an agent-based null model that follows the same rules. More specifically, using the weighted network links as the response variables, we compare the results of a linear regression model fitted to the observed data with those from synthetic data generated by an assumed agent-based null model, for example, the null model of individuals interacting at random. The sequence of model outputs over time maps out a trajectory, enabling us to see how social norms become progressively more or less important. Furthermore, we provide a method for detecting specific individuals whose behaviour is very different from others. We note that VIAPPL data has been analysed previously^10–12^, but in an aggregated form that ignored individual interactions. Moreover, although VIAPPL data is our motivating example, the proposed methodology is applicable beyond this specific setting to other social interaction/network data.

The outline of the paper is as follows. First, we describe the VIAPPL experimental environment in more detail before discussing our proposed modelling approach. We then apply the model to investigate the presence of ingroup favouritism and reciprocity, and provide a method for visualising the data based on the model. Finally, we conclude by discussing the results of the analysis and further applications for the modelling framework.

## Motivating example: VIAPPL

In this article, we focus on social interaction data collected using VIAPPL (the Virtual Interaction APPlication). We confirm that this study was carried out in accordance with all relevant ethical guidelines and regulations. The experimental materials and procedures were approved by the relevant Research Ethics Committee (Human Sciences Research Ethics Committee of the University of KwaZulu-Natal: HSS/0213/013). All participants provided written informed consent to participate in the study and for anonymised data to be collected and used for research purposes without reservation.

**Figure 1:**
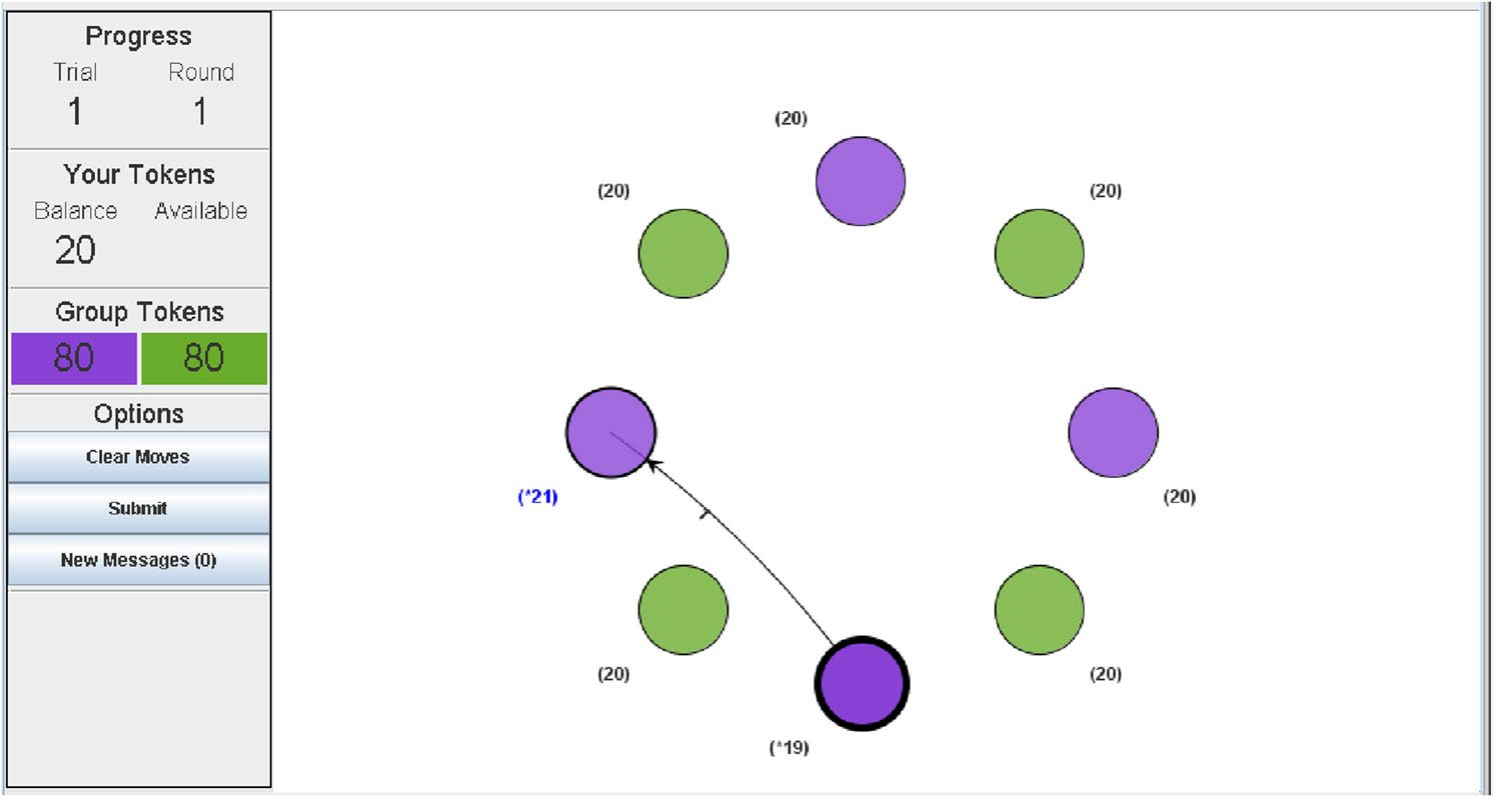
VIAPPL screen presented to a player as they select who they will allocate a token to.

**Figure 2:**
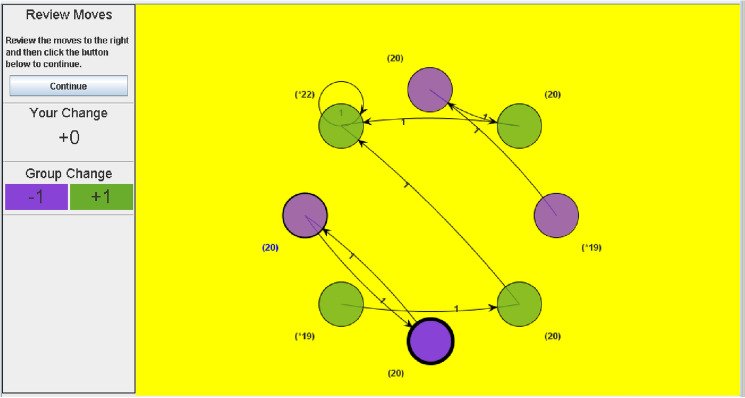
VIAPPL screen presented to players at the end of a round, displaying all token allocations from that round.

VIAPPL is a software platform for conducting experiments in social psychology. Participants of the experiments are avatars in a game-like environment and are referred to as players. They observe other players as nodes in a network, as shown in Fig. 1, and they interact by exchanging tokens over a number of rounds. In a typical study, players are randomly allocated to one of two groups at the start of the game, with group membership indicated by the node colour (purple or green). The number of tokens each player has is indicated next to their node, with group totals shown to the left of the screen; note that, in the game shown in Fig. 1, all players start with 20 tokens. A player recognises their own node by the thick black border, and selects a node to allocate a token (where they may select their own node); each player allocates one token per round. The ego player in Fig. 1, who is in the purple group, has given a token to another player in the purple group (i.e., ingroup giving), as indicated by the arrow. The token balance of the ego player is reduced by one, while the balance of the other player increases by one. Once all players have made their move, a new screen appears which displays all token allocations from that round in a directed network diagram, as in Fig. 2. Note that, in this round, the ego player has received a token from the player to whom they gave their token. An example of self-giving can be seen by the green node at the top left of the screen, as indicated by the self-directed arrow. Each player reviews the interactions of the round before the next round commences.

Many of the features in the VIAPPL platform can be modified to accommodate different experimental setups. The initial number of tokens assigned to each player need not be homogeneous, and may differ with respect to the group. Different rate-limits for token-transfer can be specified, and self-giving can be disallowed. The number of players, groups, and players per group can also be varied. Nodes may be located anywhere on the screen, not necessarily in a circular format. Nodes can also be different shapes or images, allowing players to express individuality so that they may be defined beyond their group membership. In this paper, we apply our methodology to a balanced setup like that of Fig. 1 where there are two groups, an equal number of players per group, where each player starts with the same number of tokens. However, in the games we consider, there are seven players (rather than four) per group where each individual starts with forty tokens (rather than twenty). Moreover, the game duration is forty rounds. In any case, the methodology we present can be adapted to other setups as well as to social interaction data more generally (from sources other than VIAPPL).

## Modelling approach

As described in the “Introduction” section, individual interactions are key to the formation and recreation of norms, and, thus, we model this data by considering the token exchanges between pairs of individuals as the response variable of interest. More specifically, we define $Y_{ijt}$ to be the number of tokens player *i* has *received* from player *j* up to and including round *t*. VIAPPL players give and receive tokens and are placed in groups, and, therefore, the two fundamental norms that may arise within a VIAPPL game are: reciprocation (exchanges between pairs of players) and ingroup favouritism (exchanges between players within the same group). More generally, one might define any other criterion (e.g., players preferring to give to “poorer” players) to examine whether or not it develops significantly during the course of a game, and that could then be termed a “norm”. However, in this paper, we focus on the two fundamental VIAPPL norms (reciprocation and ingroup favouritism) by considering the model

1 $$\begin{aligned} Y_{ijt} = \alpha _t + \rho _t\,Y_{jit} + \gamma _t\,G_{ij} + \varepsilon _{ijt}, \qquad i \ne j. \end{aligned}$$

where $Y_{jit}$ is the number of tokens player *i* has *given* to player *j* up to and including round *t*, $G_{ij}$ is a binary variable such that $G_{ij}=1$ indicates players *i* and *j* are in *different* groups, and $\varepsilon _{ijt}$ is an error term (whose distribution is not specified in our work). This model is only valid for $i \ne j$ since the concepts of reciprocity and ingroup favouritism are not meaningful when a player self-gives ($i = j$). The model coefficients, $\rho _t$ and $\gamma _t$, represent the reciprocity and group effects, respectively, at round *t*, while $\alpha _t$ is the intercept. Fitting the model over a sequence of *t* values allows us to discover how the game dynamics change over time. Informally, we could write the model as

$$\begin{aligned}{}&\text {``Tokens received from an individual up to round }t'' \\&\qquad = \alpha _t + \rho _t\,\text {``Tokens given to that individual up to round }t'' \\&\qquad \qquad + \gamma _t\,{\text {``Do groups differ?''}}. \end{aligned}$$

The parameters of the model can be estimated using least-squares, where we do not make any probabilistic assumptions about the data since we do not specify the distribution of the error $\varepsilon _{ijt}$. Note, in particular, that the pair $(\rho _t, \gamma _t)$ provide a useful description of the data: positive $\rho _t$ values imply reciprocity while negative values $\gamma _t$ values imply ingroup favouritism.

A small extension of Eq. (1), and one which we will consider in our analysis, includes an interaction between $Y_{jit}$ and $G_{ij}$ via

2 $$\begin{aligned} Y_{ijt} = \alpha _t + \rho _t\,Y_{jit} + \gamma _t\,G_{ij} + \delta _t\,G_{ij}\,Y_{jit} + \varepsilon _{ijt}, \qquad i \ne j, \end{aligned}$$

where $\delta _t$ adjusts the reciprocity effect in light of the group status, and vice versa, which would be useful to include if, for example, the strength of reciprocity is boosted by players being in the same group.

### Agent-based null model

There are dependencies in this type of social interaction data which invalidate classical modelling assumptions of independence. In particular, there is high connectivity between the small number of players experiencing the same game, and the number of interactions is constrained (since players each give one token per round of the game). Therefore, inferences based on the outputs of standard methods (e.g., standard errors and p values) are not likely to be valid. Moreover, our primary aim is to explore the extent to which the observed player behaviour differs from particular null models of interest, such as all players giving at random. The use of null models is quite common in network analysis^24^, and has been used in the analysis of online influence and opinion evolution^25^, but is not so prevalent in social psychology; interestingly, the approach has also been used in a range of ecology applications^26,27^.

We implement the null models of interest using agent-based models^28–30^ to generate synthetic games. As an example, the giving-at-random null is of particular interest since it represents the case where player interactions are *not* driven by the social norms of reciprocity and ingroup favouritism. In this case, the agent-based model assumes that a player has an equal probability of interacting with each player in the network in a given round. Having simulated one such synthetic game, the model of Eq. (1) is fitted to produce a random draw of estimated coefficients for the null model, say, $(\alpha ^*,\rho ^*,\gamma ^*)$. Repeating this a large number of times (we use 10,000 replicates), provides a reference distribution to which the coefficients observed on the real games can be compared; discrepancies between the real and synthetic game coefficients indicate that player behaviour differs from the null model. Besides completely random null games, we also consider semi-synthetic games where we use the observed interactions from the real data and then replace one player with a random agent (to explore the importance of that particular player) or replace two highly reciprocal players with random agents (to explore their impact on the estimated reciprocity effect). It is worth highlighting that this procedure of fitting a model to synthetic data generated from an agent-based null model is very a generic one. We use linear regression as our fitted model, which is interpretable (as described above) and will be familiar to most researchers, but, of course, other fitted models could alternatively be used.

## Results

To demonstrate our methodology, we consider data collected from 4 VIAPPL experiments, each of which had a different group of 14 participants (56 participants in total). The experiments were carried out in the University of KwaZulu-Natal, South Africa. The games each had 2 groups of 7 players, and each player started with 40 tokens. At the start of the game, players were instructed to give one token per round and were told that the game would last for 40 rounds. Since players started with 40 tokens, they could not run out of tokens at any point in the game. (Note that these games differ slightly from the example presented in Figs. 1 and 2 in that there are more players and tokens.).

### Exploratory analysis

Before applying the proposed modelling approach to the data, we first carry out some exploratory analysis to gain some initial insight. Here, we retain the *ii* data points (self-giving), but, as described above, they are removed when applying the model.

**Figure 3:**
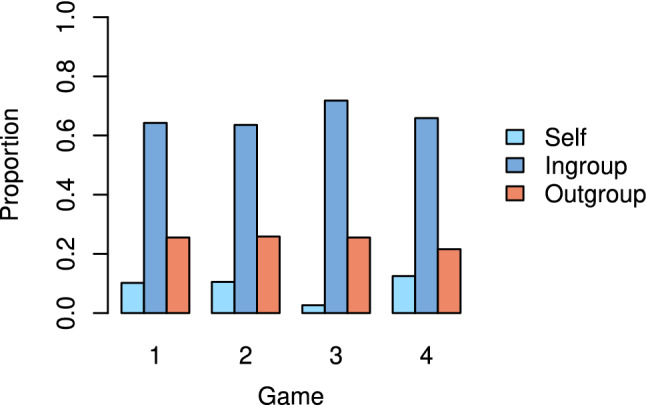
Proportion of tokens received from ingroup, outgroup and self in each game.

**Figure 4:**
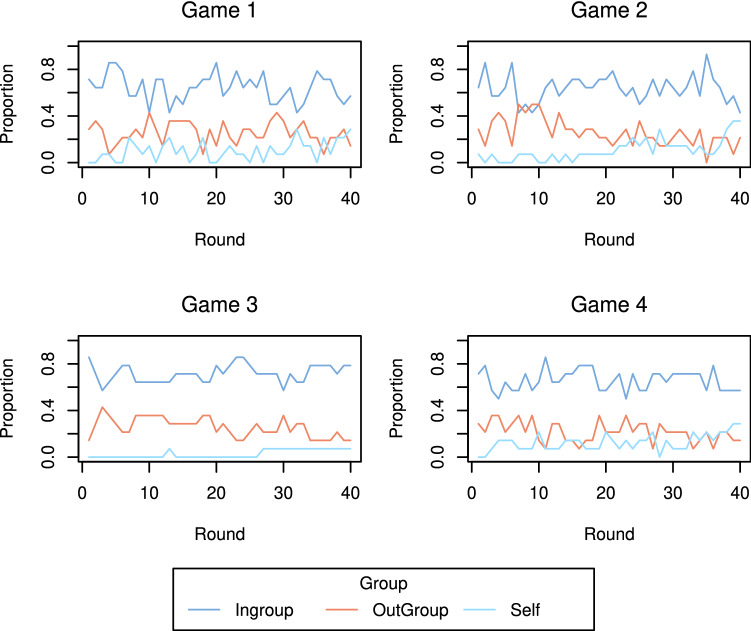
Proportion of tokens received from the ingroup, outgroup and self at each round.

Figure 3 shows the proportion of tokens players received from their ingroup, the outgroup and from themselves by the end of the game. Interestingly, players receive at least twice as many tokens on average from their ingroup as they do from their outgroup (and recall that group membership is randomly assigned). The values of each of the three proportions are remarkably similar across games, albeit game 3 has less self-giving and more ingroup giving. Figure 4 displays these proportions calculated at each round. In almost all cases, players receive more tokens from their ingroup than their outgroup. The amount of self-giving increases slightly with time in all games, and, in fact, exceeds the outgroup proportion towards the end of games 1, 2, and 4.

**Figure 5:**
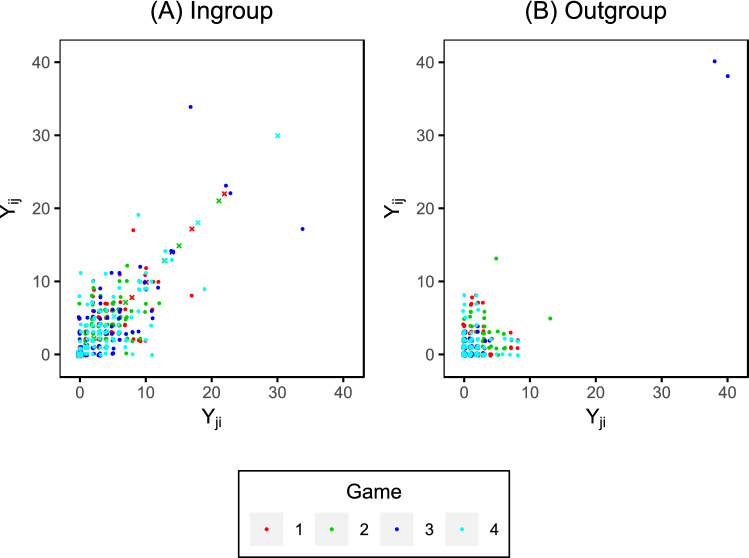
The number of tokens received is plotted against the number of tokens given for (**A**) players in the same group and (**B**) players in different groups. The $\times$ symbol indicates self-giving. The points at (38, 40) and (40, 38) in plot (**B**) correspond to two players who reciprocated for almost the entirety of the game.

The scatterplots in Fig. 5 show the relationship between the number of tokens received and the number of tokens given by the end of the game, split by group. Each point $({Y}_{ji}, {Y}_{ij})$ corresponds to a pair of players (*i*, *j*), indicating that player *i* *gave* ${Y}_{ji}$ tokens to player *j* (x-axis) and *received* ${Y}_{ij}$ tokens from player *j* (y-axis). (Note that, when referring to the whole game, we avoid writing $Y_{ij,40}$ for convenience.) The scatterplots are symmetric about the diagonal ${Y}_{ij}={Y}_{ji}$, since, for each pair of players (*i*, *j*), there are two points $({Y}_{ji},{Y}_{ij})$ and $({Y}_{ij},{Y}_{ji})$. In particular, cases of self-giving corresponds to the point (${Y}_{ii},{Y}_{ii}$) which, necessarily, appears on the diagonal in Fig. 5A (as it is an ingroup exchange). We see that points tend to be closer to the diagonal in the case of ingroup giving (Fig. 5A), indicating that players reciprocate with members of their own group. The plot corresponding to the outgroup, Fig. 5B shows points further away from the diagonal, indicating less reciprocation in general.

Table 1 displays the correlation coefficient between tokens received, ${Y}_{ij}$, and tokens given, ${Y}_{ji}$, split by the ingroup and outgroup, and by game; the closer this value is to one, the greater the reciprocity. In all games, there is a reasonably strong ingroup reciprocity, whereas outgroup reciprocity is much weaker in games 1, 2, and 4. On the other hand, in game 3, the outgroup reciprocity is very strong. However, this high correlation coefficient is mainly driven by two players with unusual behaviour, and removing their token exchanges reduces the correlation to 0.08. These two players, from different groups, formed a reciprocal relationship in which they exchanged tokens in almost all of the rounds; this is quite unlike what we observe in other players across all games. Their token exchanges are clearly visible in the top-right corner of Fig. 5B, lying far away from all other points (even when compared to the ingroup setting of Fig. 5A); later in our analysis, these two players are also identified as being highly unusual based on the model.

**Table 1:** Correlation between ${Y}_{ij}$ and ${Y}_{ji}$ for the ingroup and the outgroup.

	Game			
	1	2	3	4
Ingroup	0.68	0.69	0.80	0.73
Outgroup	0.09	0.33	0.97	− 0.06

### Model for the full game

We apply the linear model, described in “Modelling approach” section, to the full game, i.e., exchanges up to, and including, the final round ($t=40$). The response, $Y_{ij}$, is the number of tokens player *i* received from player *j* over the course of the game. We consider four models which are special cases of Eq. (2):

- reciprocity effect only ($\gamma = \delta = 0$),
- group effect only ($\rho = \delta = 0$),
- additive reciprocity and group effects ($\delta =0$), and
- interacting reciprocity and group effects.

Table 2 displays the $R^2$ values for these four models in each of the four games, where we have used $R^2$ as it is a generic measure of explained variation that aligns with the least-squares estimation procedure. Note that the highest $R^2$ values are seen in game 3, but this is driven by the two anomalous players mentioned earlier. Looking at the other games, we see that the models with additive effects have larger $R^2$ values than the models with only one of the two effects, whereas the inclusion of the interaction effect increases the $R^2$ to a much lesser extent.

**Table 2:** $R^2$ for models for the full game.

Effect type	Model structure	Game			
		1	2	3	4
Reciprocity	$\rho \,Y_{ji}$	0.22	0.25	0.78	0.26
Group	$\gamma \,G_{ij}$	0.24	0.29	0.08	0.23
Additive	$\rho \,Y_{ji} + \gamma \,G_{ij}$	0.31	0.35	0.78	0.33
Interaction	$\rho \,Y_{ji} + \gamma \,G_{ij} + \delta \,G_{ij}*Y_{ji}$	0.33	0.35	0.79	0.37

**Figure 6:**
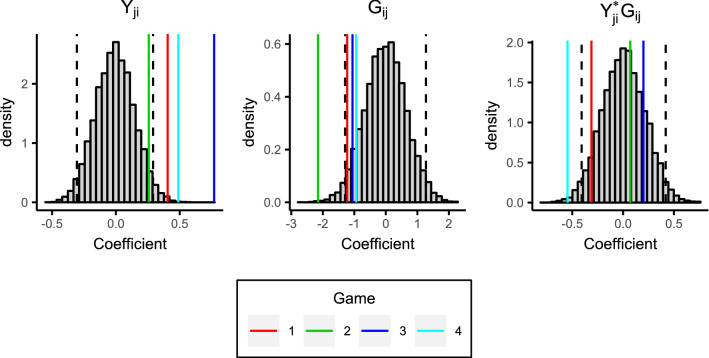
Coefficients for the interaction effects models. Game coefficients are the vertical coloured lines. The histograms show the model coefficients from synthetic giving-at-random null games. $95\%$ of values lie between the black dashed lines.

**Figure 7:**
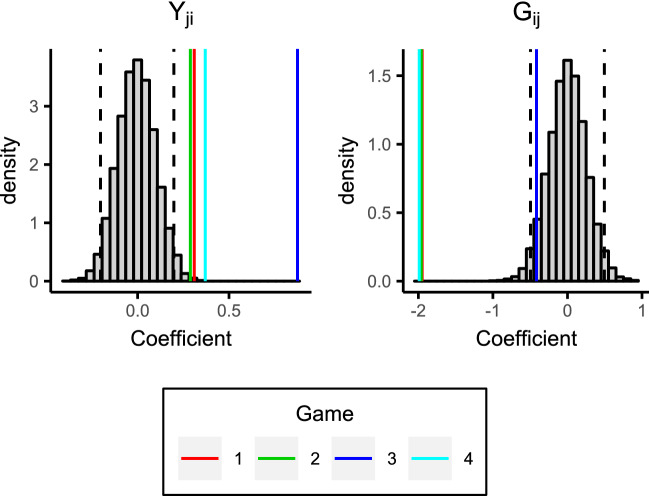
Coefficients for the additive effects models. Game coefficients are the vertical coloured lines. The histograms show the model coefficients from synthetic giving-at-random null games. $95\%$ of values lie between the black dashed lines.

Figure 6 displays the coefficients for the interaction effects models, along with histograms of the coefficients obtained from the synthetic giving-at-random null games. Note that the interaction term is within the $95\%$ bounds for games 1, 2, and, 3, and only just outside of these bounds for game 4, i.e., the interaction effect is not strong as was also suggested by Table 2. We therefore consider the additive effects models in more detail; Fig. 7 displays the coefficients against the reference null distributions, and Table 3 provides the numeric values of these coefficients along with p values computed based on the null distribution. It is clear the group effect is strong and highly significant statistically, with players in games 1, 2, and 4 receiving approximately two fewer tokens on average from outgroup members than ingroup members. The reciprocity effect is not as strong, but it is still highly significant for all four games; its positive value indicates that players receive more tokens from players to whom they give tokens. Again it is clear that the behaviour in game 3 differs from the others, with a group effect which is non-significant (at the $5\%$ level), and a much stronger reciprocity effect.

**Table 3:** Coefficients for the additive effects models.

	Game			
	1	2	3	4
Intercept	2.960 (0.787)	3.010 (0.656)	0.600 (< 0.001)	2.770 (0.757)
${{Y}}_{{ji}}$	0.310 (0.001)	0.290 (0.004)	0.870 (< 0.001)	0.370 (< 0.001)
${{G}}_{{ij}}$	− 1.950 (< 0.001)	− 1.960 (< 0.001)	− 0.420 (0.106)	− 1.990 (< 0.001)

p values are in brackets next to the coefficients.

### Model for each round

**Figure 8:**
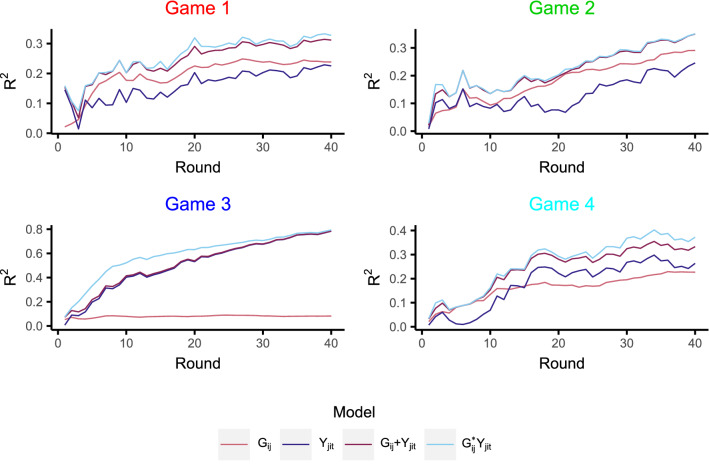
$R^2$ for each model at each round.

While the previous section focused on the exchanges between players by the end of the game, i.e., $t=40$, we now fit the various models, but at all time points $t\in \{1,2,\ldots ,40\}$ to analyse the evolution of the effects over time. Figure 8 displays the $R^2$ for each model at each round. We see that the $R^2$ values are generally increasing over time, suggesting that the behaviour becomes more predictable, perhaps as players settle into some normative behaviour. It is also clear that, over essentially all time points, the additive effects models improve on the single reciprocity and group effects models, while the interaction effects models are only slightly better than the additive effects models; the exception to this is game 3 (the unusual game) in the earlier rounds where the interaction effects models do appear to improve the fit (albeit this is not the case in the latter rounds).

**Figure 9:**
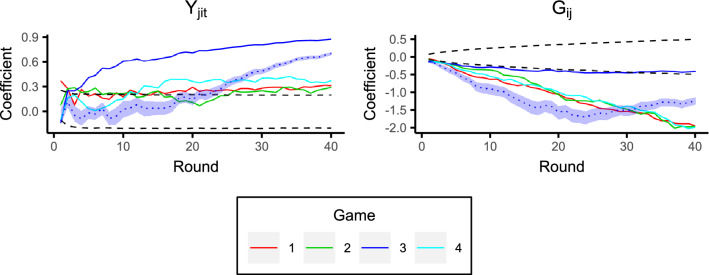
Coefficients for the additive effects models at each round. Solid coloured lines are the game coefficients. Black dashed lines are the $95\%$ bands from the synthetic giving-at-random null games. The dotted line is the average coefficient from semi-synthetic data based on game 3 (explained in the “Game 3” section), and the surrounding shaded region displays the $95\%$ bounds.

The coefficients for the additive effects models over each round are shown in Fig. 9. For games 1, 2, and 4, the group effects are significant at almost all rounds, i.e., the norm of giving to the ingroup is apparent from the outset. Moreover, this ingroup giving behaviour strengthens over time, as evidenced by the increasing discrepancy between the group coefficients and the null distribution ($95\%$ bounds) as the rounds progress. In contrast, the reciprocity effect is non-significant in the earlier rounds, indicating that it takes longer for individuals to build reciprocal links. Indeed, recall from Fig. 5 that individuals reciprocate more with individuals from the ingroup. This, in combination with the fact that the group effect is established much earlier in the game, perhaps suggests that the group membership provides a context for reciprocal links to later develop. The coefficients, and their trajectories over time, are remarkably similar across these three games (1, 2, and 4), particularly the group effect, i.e., there is repeatability in the dynamics over different groups of participants. However, game 3 is quite different from the other games with a much larger reciprocity effect, and a much smaller (non-significant) group effect (which we consider in more detail in the next section).

### Game 3

Up to now, we have not explicitly referred to the identity of a specific player in a given experiment, beyond pointing out that an unusual pair of interactions exists in game 3 (which was clearly visible in Fig. 5B). Of course, there are 14 individuals in each game, and, therefore, we have (arbitrarily) assigned each player a unique identification number $i \in \{1,\ldots ,14\}$ to which they can now be referred. In game 3, player 3 gave 38 (out of a possible 40) tokens to player 8, while player 8 gave 40 tokens to player 3. This level of reciprocity was far higher than between any other pairs, both in game 3 and the other games. In this section, we investigate the extent to which these two players contributed to the large reciprocity effect for that game, and, indeed, the small group effect (since these players are in different groups).

To examine the influence of players 3 and 8 on the estimated model coefficients, we create semi-synthetic data in which these players are replaced with null players who give at random. This data was created by artificially replaying the game, keeping all token exchanges as per the originally observed data, apart from the tokens given by players 3 and 8, which are instead allocated at random. This has the effect of altering only the values of $Y_{i,3,t}$ and $Y_{i,8,t}$, i.e., in the real data, $Y_{i,8,t}$ equals *t* for $i=3$ and zero otherwise, whereas, in the semi-synthetic data, $Y_{i,8,t}$ equals *t*/14 on average $\forall i$; similarly, in the semi-synthetic data, $Y_{i,3,t}$ equals *t*/14 on average $\forall i$.

In total, we generated 10,000 semi-synthetic datasets, and applied the additive effects model to each. This yields a distribution for the model coefficients with the unusual reciprocal behaviour of players 3 and 8 removed. The average coefficients from this distribution along with $95\%$ bounds are shown in Fig. 9 (dotted line and shaded area). Indeed, compared to the real game 3, the reciprocity effect is reduced, but still becomes larger than that of the other games; this suggests that an unusual level of reciprocity exists in game 3 even having removed the influence of players 3 and 8. Note that, in Fig. 5A, there are points in positions (17, 4) and (34, 7) corresponding to players 1 and 5, and points in positions (23, 2) and (22, 3) corresponding to players 2 and 12; they are much nearer to the top right corner than any other (non-self-giving) points, indicating sustained reciprocity. These two reciprocal pairs are not as unusual as the player 3 and player 8 pairing, since they correspond to players in the same group, and, as we have seen, ingroup giving is normative behaviour. For this reason, the group effect in the semi-synthetic game 3 is still very strong. However, it does reduce in later rounds as the reciprocity effect becomes stronger in this game.

### Influence metric

Players 3 and 8 in game 3 were initially identified as being unusual through the exploratory analysis, i.e., they are clear outliers in Fig. 5B, and their impact was also clearly visible in the model coefficients from Fig. 9. Because of this, it was natural to investigate these two specific players in that specific game in more detail. However, we now develop a more systematic, model-based approach for identifying anomalies by way of their influence on the model coefficients, which can be applied in any of the games. A standard statistical technique for identifying influential observations is to remove an observation from the dataset, refit the model to this reduced dataset, and compute the difference between the new and original coefficients, so-called “dfbetas”^31^; large changes in the coefficients signify influential observations. In our context, for example, a large change in the reciprocity coefficient identifies a specific player who reciprocated more or less than average.

In our context of social interaction data, however, simple removal of observations changes the structure of the data due to the interrelatedness of these observations. For example, removing $Y_{ji,{40}}$ leads to player *i* having given $40 - Y_{ji,{40}}$ tokens by the end of the game, whereas all others give 40 ; removing the set of all tokens given by player *i*, $(Y_{1,i},\ldots ,Y_{14,i})$, creates a non-player who still received tokens; removing player *i* entirely creates imbalance in the group sizes, and, furthermore, all tokens related to this individual (giving and receiving) become unaccounted for. These removals alter the data structure, which is why, in examining the joint influence of players 3 and 8 in game 3, we did not simply *remove* these players but, rather, *replaced* them with agent-based players who give tokens at random. This maintains the structure of the data where token exchanges are redistributed. Thus, in order to determine the influence of a single player on the model coefficients, we use the same approach here but with an individual player, rather than a pair, i.e., we replace a single player with an agent-based player who gives at random. It should be noted, however, that we are not aiming to discover how a random-giver influences the dynamics of a game. (But that is an interesting research direction requiring a non-human random-giving player to be inserted into a live game a priori.) Rather, we wish to determine the impact of a given player on the estimated model coefficients (in the spirit of “dfbetas”), but where we avoid the data imbalances that would arise from a simple removal as previously described.

Analogous to the previous section, we create semi-synthetic games where everything remains as per the observed data apart from the actions of player *i* who is replaced by a null player who gives at random (and note that we are now not focused only on game 3). For the *k*th semi-synthetic game, we fit the model to obtain the altered coefficients ${\hat{\rho }}_{k,t}^{-i}$ and ${\hat{\gamma }}_{k,t}^{-i}$ for $t=1,\ldots 40$, where the superscript “$-i$” indicates that the influence of player *i* is removed. These coefficients map out a curve that could be compared to those of Fig. 9. However, it is not practical to view 14 altered coefficient curves (one for each player in the game) for both coefficients across each of the four games. We therefore compute the distances between altered and original curves using an $\ell _1$ norm for each synthetic game,

$$\begin{aligned} \Vert {\hat{\rho }}-{\hat{\rho }}_{k}^{-i}\Vert _{1}, \quad \qquad \Vert {\hat{\gamma }}-{\hat{\gamma }}_{k}^{-i}\Vert _{1}, \end{aligned}$$

where ${\hat{\rho }} = ({\hat{\rho }}_{1},\ldots ,{\hat{\rho }}_{40})$ and ${\hat{\gamma }} = ({\hat{\gamma }}_{1},\ldots ,{\hat{\gamma }}_{40})$ are the vectors of original coefficients, and ${\hat{\rho }}_k^{-i} = ({\hat{\rho }}_{k,1}^{-i},\ldots ,{\hat{\rho }}_{k,40}^{-i})$ and ${\hat{\gamma }}_k^{-i} = ({\hat{\gamma }}_{k,1}^{-i},\ldots ,{\hat{\gamma }}_{k,40}^{-i})$ are the vectors of altered coefficients for synthetic game *k*, and $\Vert x \Vert _{1} = |x_1| + |x_2| + \cdots |x_{40}|$ is the $\ell _1$ norm for a vector *x* of length 40. Then, we average these over the replicate synthetic games to obtain the influence metrics,

3 $$\begin{aligned} d\rho _{i} = \frac{1}{N}\sum _{k=1}^{N}\Vert {\hat{\rho }}-{\hat{\rho }}_{k}^{-i}\Vert _{1}, \quad \qquad d\gamma _{i} = \frac{1}{N}\sum _{k=1}^{N}\Vert {\hat{\gamma }}-{\hat{\gamma }}_{k}^{-i}\Vert _{1}, \end{aligned}$$

where *N* is the number of simulated agent-based game replicates; we use $N=10{,}000$. In order to make these metrics more comparable, we standardise them by dividing by the average value across all players in the game,

4 $$\begin{aligned} \frac{d\rho _{i}}{\sum _{i=1}^{14} d\rho _{i} / 14}, \quad \qquad \frac{d\gamma _{i}}{\sum _{i=1}^{14} d\gamma _{i} / 14}. \end{aligned}$$

Values greater than 1 indicate player *i* is more influential than the average player.

These values are shown in Table 4, where we also highlight values greater than 2 as these correspond to players whose influence is more than double the average. While there are players with influence scores greater than 2 in all games (only just in some cases), there are also players with scores much larger than this. In game 3, players 3 and 8 have influence scores greater than 5 for both the reciprocity and group effects, which indicates that their behaviour strongly influenced the estimated model coefficients; this is in line with our findings in the previous section. These players were far more influential than players in any of the other games. Interestingly, there are two players in game 4 with quite high influence scores. Player 6 has a large influence score for the group effect. It turns out that this player gave no tokens to outgroup players, and, although ingroup giving is normative, it is unusual to have avoided the outgroup entirely (in game 4, players gave 8.6 tokens to outgroup players on average). Player 7 has large influence scores for both effects, albeit more for the reciprocity effect than the group effect. This player reciprocated 35 tokens throughout the game, of which 34 were with ingroup players (in game 4, players reciprocated 20.6 tokens on average).

**Table 4:** Standardised influence metrics.

Player	Game							
	1		2		3		4	
	$Y_{ji}$	$G_{ij}$	$Y_{ji}$	$G_{ij}$	$Y_{ji}$	$G_{ij}$	$Y_{ji}$	$G_{ij}$
1	0.28	0.26	0.69	0.41	0.73	0.61	0.39	0.27
2	0.91	1.30	0.59	0.62	0.50	0.42	1.63	0.43
3	2.01*	0.44	0.51	0.35	5.19*	5.44*	1.52	1.13
4	1.39	0.70	0.46	1.51	0.33	0.16	0.53	1.18
5	0.43	0.80	0.42	1.21	0.39	0.59	0.58	0.21
6	0.91	1.65	1.22	1.04	0.24	0.18	0.69	3.71*
7	1.38	0.89	1.62	0.94	0.18	0.11	3.76*	2.72*
8	1.10	0.32	1.05	1.48	5.09*	5.46*	0.27	0.21
9	0.44	0.85	1.72	0.85	0.15	0.21	0.37	0.33
10	0.25	0.80	1.23	1.68	0.16	0.16	0.84	1.27
11	0.66	0.85	0.61	0.63	0.11	0.07	1.53	0.84
12	2.52*	1.96	2.23*	0.80	0.56	0.34	0.42	0.63
13	0.59	1.85	0.96	1.69	0.09	0.05	0.37	0.32
14	1.11	1.32	0.69	0.79	0.28	0.21	1.10	0.74

Values larger than 2 are marked with a * to indicate players with more influence on the model.

### Network visualisation

**Figure 10:**
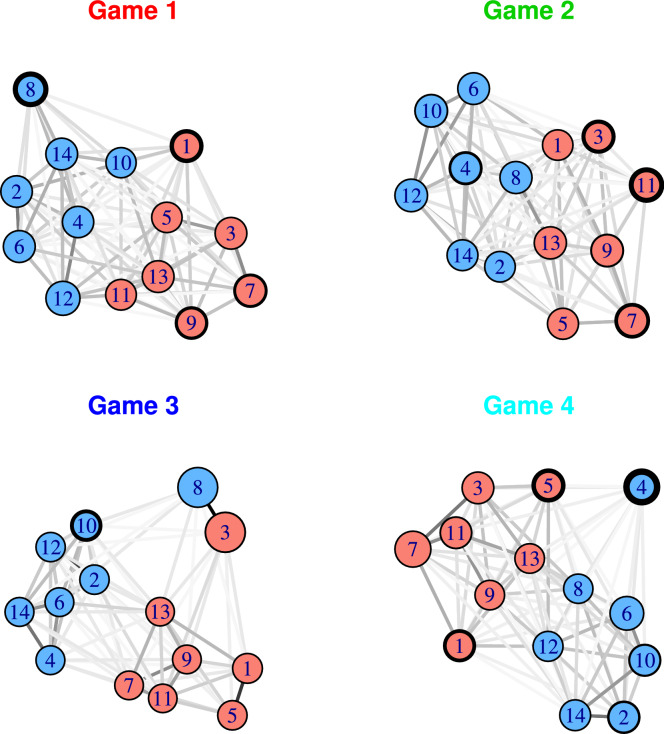
The network of players for each game. The node colour indicates the group while the node size relates to size of the influence scores from Table 4 (larger nodes indicate higher scores). Edges between nodes are drawn if players exchanged tokens, where the edge colour corresponds to the total number of exchanges between the players in either direction (darker edges correspond to more exchanges). The size of the border around a node indicates the level of self-giving for that player.

Figure 9 provided a visualisation of the game dynamics over time, aggregated over individuals through model coefficients. We now provide a complementary network diagram visualisation in Fig. 10 which provides an alternative view in terms of how individual player’s contribute to the aggregate normative behaviour. This is a weighted network produced based on the total number of token exchanges between pairs of players (in either direction) by the end of the game (albeit such diagrams could be produced at any time point in the game).

From the network diagram, we get a sense of which specific players reciprocated with each other, the level of ingroup or outgroup giving, and how unusual players are based on their influence score. The network layout was generated using the Fruchterman–Reingold layout algorithm in the igraph package in R^32,33^, a force-directed algorithm that locates nodes more closely together if their edge weight is larger.

As we would expect based on our earlier analysis, there is a clear split between the two groups, showing the tendency of players to interact more with members of their ingroup. Players who are located more centrally in the network interacted with both groups, e.g., players 10 and 11 in game 1, whereas players at the periphery of the network interacted more exclusively with their ingroup, e.g., players 2 and 3 in game 1. Of course, players 3 and 8 in game 3 stand out clearly, both due to their node size (influence score), and the fact that they are they are located further away from other nodes. This is due to the fact that they essentially only interacted with each other, however there are still weak links with other players meaning that, throughout the game, other players did interact with them to some extent. Player 4 in game 4 is also quite distinctive. This player self-gave tokens 30 times during the game (visible in the thick border), and, much like players 3 and 8, is located further away from other nodes due to the lower level of interaction with other players.

## Discussion

We have introduced a modelling approach suitable for online interactive social experiments that uses a directed network to compare with suitable null models. We presented a number of examples using the Virtual Interaction APPLication. VIAPPL data has been analysed previously, but in an aggregated form which did not previously allow the effect of individual interactions to be studied^10–12^. Our approach indicates that ingroup favouritism is a prominent normative behaviour that emerges right from the outset of the game; this is in agreement with the previous VIAPPL research. We have additionally found that players have a tendency to reciprocate, and this behaviour becomes more pronounced as the game progresses, i.e., it takes time for a reciprocal relationship to develop, whereas ingroup favouritism is more immediate. The ability to detect reciprocation is only possible by modelling the interactions between individuals, which we have done in this paper.

Interestingly, the estimated group and reciprocity effects are quite similar across 3 of the 4 games. However, one of the games produced quite different results from the rest. We showed that this was mainly due to two players from different groups who reciprocated with each other for most rounds of the game (albeit there were other high-reciprocating players in that game). Our model-based influence metric identified these individuals as being unusual, i.e., their behaviour (high reciprocation with an outgroup member) was much different from the average behaviour in that game. When these two players were removed from the data, the results of the model were much more similar to results from the other games. Our visualisation of the network of players complements the output from the linear model, as it indicates the contribution of individual players to the overall dynamics estimated by the model.

We note that our suggested influence metric (for detecting individuals differing from the norm) could be used to detect anomalous individuals in various settings. One topical example of this type of application is bot detection on Twitter, where the focus is on determining user accounts that display unusual behavioural patterns^34^. It would be interesting to apply the approach to such data to determine whether or not bot accounts are indeed identified, and visualise the network as per Fig. 10; such information could complement other methods currently employed for bot identification^35^.

In this paper, we have considered a model that can capture reciprocity and ingroup giving, as these are the fundamental VIAPPL behaviours. However, the model could be extended to include additional predictor variables via

$$\begin{aligned} Y_{ijt} = \alpha + \rho \,Y_{jit} + \gamma \,G_{ij} + \delta \,G_{ij}\,Y_{jit} + {\beta ^T} X_{ijt} + \varepsilon _{ijt}, \end{aligned}$$

where $X_{ijt}$ is a vector of predictors and $\beta$ is a corresponding vector of regression coefficients. These additional predictors could relate to the individual, such as age or gender; to aspects of the game’s history, such as the number of tokens that player *j* received from player *i* over some particular window of time, $Y_{ji{t_2}}-Y_{ji{t_1}}$; or to conditions of the experimental setup (if multiple setups are considered in the same analysis), such as whether or not individuals started with the same number of tokens. More generally, interaction terms could be introduced between elements of $X_{ijt}$ and $Y_{jit}$, $G_{ij}$, or other elements of $X_{ijt}$. Clearly, the use of such predictors within this model would allow us to gain further insight into VIAPPL behaviours, and this will be considered in our future work.

Although VIAPPL is our motivating example and focus for this paper, the methodology we have proposed can be used for any kind of social interaction data; of course, these interactions need not be “token exchange” (which is just the social interaction mechanism within the VIAPPL platform). Our proposed approach can be applied irrespective of the constraints and interdepencies in the data—which is useful when standard modelling assumptions are not met. The use of agent-based null models makes this approach particularly suited to scenarios where the data is produced according to some rule-based process, for example, in iterated prisoner’s dilemma experiments in game theory^36^. However, this does not prevent its use in other settings^28–30^ once a suitable agent-based model is defined for the scenario under study. Therefore, we anticipate that the general approach taken in this paper (of comparing models fitted to real and synthetic agent-based data) could be applied in a variety of application areas.

## Supplementary Information

Supplementary Information.

## Data Availability

The anonymised data considered in this article are included in the supplementary material.
